# Perceptions of Coach–Athlete Relationship Are More Important to Coaches than Athletes in Predicting Dyadic Coping and Stress Appraisals: An Actor–Partner Independence Mediation Model

**DOI:** 10.3389/fpsyg.2016.00447

**Published:** 2016-03-29

**Authors:** Adam R. Nicholls, John L. Perry

**Affiliations:** Department of Sport, Health and Exercise Science, University of HullHull, UK

**Keywords:** dyads, relationships, systemic-transactional model, coping, threats, challenges

## Abstract

Most attempts to manage stress involve at least one other person, yet coping studies in sport tend to report an athlete’s individual coping strategies. There is a limited understanding of coping involving other people, particularly within sport, despite athletes potentially spending a lot of time with other people, such as their coach. Guided by the systemic-transactional model of stress and coping among couples (Bodenmann, 1995), from relationship psychology, we assessed dyadic coping, perceptions of relationship quality, and primary stress appraisals of challenge and threat among 158 coach–athlete dyads (*n* = 277 participants). The athletes competed at amateur (*n* = 123), semi-professional (*n* = 31), or professional levels (*n* = 4). Coaches and athletes from the same dyad completed a measure of dyadic coping, coach–athlete relationship, and stress appraisals. We tested an Actor–Partner Interdependence Mediation Model to account for the non-independence of dyadic data. These actor–partner analyses revealed differences between athletes and coaches. Although the actor effects were relatively large compared to partner effects, perceptions of relationship quality demonstrated little impact on athletes. The mediating role of relationship quality was broadly as important as dyadic coping for coaches. These findings provide an insight in to how coach–athlete dyads interact to manage stress and indicate that relationship quality is of particular importance for coaches, but less important for athletes. In order to improve perceptions of relationship quality among coaches and athletes, interventions could be developed to foster positive dyadic coping among both coaches and athletes, which may also impact upon stress appraisals of challenge and threat.

## Introduction

Participating in sport can be stressful (see Arnold and Fletcher, 2012 for a review), so it is important that athletes cope effectively with any stress encountered. Coping is a self-regulatory mechanism used to alleviate stress, and involves conscious cognitive and physical attempts to manage internal or external demands that have been appraised as taxing a person’s resources (Lazarus and Folkman, 1984). The coping literature is awash with assessments of individual athlete’s attempts to cope with stress (Tamminen and Gaudreau, 2014; Crocker et al., 2015), despite most stressful incidents involving at least one other person (Folkman, 2009). Indeed, dyadic accounts of appraisals and coping, which explore how two people within the same stressful incident evaluate stress and subsequently interact to cope are therefore needed (Folkman, 2009; Herzberg, 2013). Due to the nature of sport, athletes and coaches are likely to be involved in the same stressful encounters (i.e., competitive performances or training sessions), yet little is known about how coaches and athletes cope together and how this may be associated with relationship quality or stress appraisals. The purpose of this paper was to assess an *a priori* model, guided by Bodenmann’s (1995) systemic-transactional model of stress and coping among couples and Ledermann et al.’s (2011) Actor–Partner Interdependence Mediation Model (APIMeM), which included dyadic coping, perceptions of relationship quality, and appraisals of challenge and threat among coach–athlete dyads.

### Dyadic Coping

A partnership that operates in many sports involves a coach and an athlete (Jackson et al., 2010). Indeed, Jackson et al. (2010) suggested that coach–athlete dyads are highly important given the amount of time coaches and athletes spend together. Furthermore, coach–athlete interactions also influence technical and physical competencies (Jowett and Poczwardowski, 2007), in addition to being related to psychological constructs such as coping (Nicholls et al., 2016a). To date, however, there are no published accounts of dyadic coping between a coach and his or her athlete. Essentially, dyadic coping relates to the way in which a couple interacts to cope (Berg and Upchurch, 2007). The primary purpose of dyadic coping is to reduce stress for both members (Bodenmann, 1995, 2005). There are some similarities between dyadic coping and a construct previously examined in the sport psychology literature, namely, social support. Both constructs are associated with stress reducing qualities (i.e., Freeman and Rees, 2008; Rottmann et al., 2015). Nevertheless, there are key conceptual differences between dyadic coping and social support. For example, dyadic coping is exclusively concerned with the way a couple interact to cope, whereas social support is much broader and includes support relating to boosting pleasant emotions or esteem, providing informational advice, or practical assistance (Freeman et al., 2014). As such, social support may be provided in the absence of stress (e.g., a coach providing tactical advice), whereas dyadic coping only occurs under conditions of stress. Furthermore, an athlete may receive social support from a variety of different individuals (e.g., coach, spouse, sibling, or peer), whereas dyadic coping refers to the interaction between two people.

Bodenmann (1995, 2005) proposed the systemic-transactional model of stress and coping among couples to explain dyadic coping, which is grounded in Lazarus and Folkman’s (1984) transactional model of stress and coping. It is important to note that Lazarus and Folkman (1984) conceived coping at the dispositional and process levels. In particular, they argued that dispositional coping (i.e., how people normally cope) represents the structure of coping and that “structure and process are both necessary for an understanding of coping” (p. 298). Assessing coping at the dispositional level represents an accurate method of assessing trends in behavior over a long period of time (Fleeson, 2005) and may also reveal habitual or generalized patterns of coping behavior that process assessments fail to capture (Hurst et al., 2011). This is paramount when very little is known about a particular type of coping (e.g., dyadic coping among a coach and his or her athlete) or its relationship with other constructs (e.g., perceptions of relation quality among coach–athlete dyads). There is an emerging trend of assessing coping at the dispositional level within the sport psychology literature to assess how coping is related to constructs such as cognitive-social maturity (Nicholls et al., 2013), emotional maturity (Nicholls et al., 2015), or behavioral engagement (Nicholls et al., 2016b).

Dyadic coping is triggered when one member of the dyad communicates stress to the other via verbal or non-verbal communications, with the other partner responding with some form of dyadic coping (Bodenmann, 1995, 2005). As such, Bodenmann argued that dyadic coping is interactive and reciprocal. Bodenmann (2005) distinguished between positive and negative types of dyadic coping. Positive dyadic coping includes three distinct types of coping: supportive dyadic coping (i.e., one partner helping the other in his or her coping efforts, such as providing practical advice or empathy), delegated dyadic coping (i.e., one person assuming responsibility of different tasks to reduce the others person’s workload), and common dyadic coping (i.e., both partners partaking in the same strategies together, such as relaxing or problem solving together). Negative dyadic coping involves hostile, ambivalent, or superficial responses to the other person and represents support that is insincere or unwillingly provided (Rottmann et al., 2015).

Although scholars are yet to examine dyadic coping between coaches and athletes, there is an emerging body of dyadic coping within the relationship literature, among married couples. For example, dyadic coping was a stronger predictor of relationship satisfaction than individual coping (Herzberg, 2013). In another study, Rottmann et al. (2015) examined dyadic coping, and thus how couples interact when the female member was diagnosed with breast cancer. Negative dyadic coping was adversely associated with outcomes for both partners, whereas common dyadic coping was associated with superior relationship quality and fewer depressive symptoms. As such, it appears that dyadic coping may influence the relationship quality between two people. Within a sport setting, Tamminen et al. (2016) demonstrated that parents influenced their children’s attempts to cope, but little is known about how dyadic coping may be associated with relationship quality among parent–athlete or coach–athlete dyads.

### Coach–Athlete Relationship

The coach–athlete relationship refers to all situations in which a coach and athlete’s feelings, thoughts, and behaviors are inter-related (Jowett and Cockerill, 2003). The most frequently cited coach–athlete conceptual model in the sport psychology literature is Jowett’s (2007) 3 + 1 Cs framework. This includes four constructs: closeness (i.e., how much the coach and the athlete care, support, and value on another), commitment (i.e., the extent to which the coach and/or athlete intend to maintain their relationship together), complementarity (i.e., the extent to which the behaviors of the coach and athlete correspond to each other), and finally, co-orientation (i.e., whether the coach and athlete have established common views regarding how the athlete may progress in his or her sport).

When the subcomponents of the 3 + 1 Cs model are combined, they represent perceptions of the overall relationship quality between a coach and an athlete (Lafrenière et al., 2011). A strong coach–athlete relationship is linked to enhanced performance (Jowett and Cockerill, 2003), happiness (Lafrenière et al., 2011), and superior self-concept (Jowett and Cramer, 2010). Although there are a number of adaptive outcomes of a stronger coach–athlete relationship, at the present time, little is known about how dyadic coping may be associated with the coach–athlete relationship. As dyadic coping includes interactions between two people, it is plausible that this form of coping will be related to relationship quality. Further, little is known about whether dyadic coping or indeed the coach–athlete relationship is associated with stress appraisals. Given that the coach–athlete relationship is related to emotions (i.e., Lafrenière et al., 2011), which are generated by appraisals, and that stress appraisals are associated with individual athlete’s attempt to cope (Nicholls et al., 2014), it is possible that both the coach–athlete relationship and dyadic coping will be related to stress appraisals. Research is required to test whether there is an association between dyadic coping and appraisals experienced within the same dyad, in order to assess this assertion.

### Stress Appraisals

The way in which a person evaluates the significance of a situation in regards to his or her personal goals, which might be endangered is known as stress appraisal (Lazarus, 1999). Athletes can anticipate either a loss or gain occurring (Lazarus, 2000). Anticipated losses, such sustaining an injury or losing an upcoming match, can be referred to as threat appraisals. Alternatively, if an athlete anticipates some form of gain such as impressing a selector or winning a monetary award, this would be considered a challenge appraisal (Lazarus, 2000). The concepts of challenge and threat within Lazarus’ (2000) conceptual model are similar to those within Blascovich’s (2008) biopsychosocial model (BPSM) of challenge and threat states, although Blascovich also identified physiological differences (i.e., heart-rate, cardiac output, and total peripheral resistance) between these two states.

Guided by the BPSM (Blascovich, 2008), scholars recently explored the implications of challenge and threat perceptions among athletes. Challenge states were associated with superior performance, less anxiety, and conscious processing than threat states (Moore et al., 2013; Turner et al., 2013). Furthermore, appraisals of challenge and threat states can be manipulated in order to maximize performance. In particular, Moore et al. (2015) employed arousal re-appraisal training to transform threat into challenge states, which yielded performance improvements. Understanding more about the antecedents of challenge and threat appraisals, such as dyadic coping and the coach–athlete relationship quality, may provide psychologists with additional mechanisms to manipulate the occurrence of challenge states, other than those tested by Moore et al. (2015).

### Summary and Hypotheses

We hypothesized a positive association between positive dyadic coping and relationship quality, but a negative path between negative dyadic coping and relationship quality. This is because scholars previously reported an association between positive dyadic and relationship quality, whereas negative dyadic coping was negatively associated with relationship quality among couples dealing with breast cancer (Rottmann et al., 2015). We also predicted a positive path from relationship quality to challenge appraisals, but a negative path from relationship quality to threat appraisals. Lafrenière et al. (2011) revealed that relationship quality was associated with happiness, which is a consequence of a gain appraisal (Lazarus, 2000). It is acknowledged that appraisals are usually modeled to precede coping in many studies (i.e., Nicholls et al., 2012, 2014), whereas appraisals were modeled after dyadic coping in the present study. Conceptually, Lazarus and Folkman (1984) viewed stress and coping as a reciprocal and dynamic constructs. As such Lazarus and Folkman (1984) theorized that appraisals generate coping, in addition to coping influencing subsequent stress appraisals. This is in agreement with Bodenmann’s (1995) model of systemic transactional coping, which included appraisals before and after dyadic coping. We hypothesized positive paths between positive dyadic coping and challenge, along with negative dyadic coping and threat, but negative paths between positive dyadic coping and threat, in addition to negative dyadic coping and challenge. These hypotheses are based on Bodenmann (1995, 2005) and Lazarus and Folkman’s (1984) assertions that positive coping facilitates challenge states, whereas the negative dyadic coping would be considered less helpful and therefore generate threat states. Researchers from the sport literature found a link between adaptive forms of coping and challenge appraisals, whereas less adaptive forms of coping are associated with threat appraisals (e.g., Nicholls et al., 2012). As such, it is plausible that dyadic coping and appraisals would be related.

To explore the main dyadic effects, we tested an Actor–Partner Interdependence Model (APIM) to account for the non-independence of dyadic data (Kenny, 1996). An APIM is able to simultaneously estimate the impact of actor effects (horizontal) within a group and partner effects (diagonal) from one group on another. Typically, APIMs contain predictor and outcome variables. We hypothesized, however, that the relationships between our predictor (dyadic coping) and outcome (stress appraisal) would be mediated by perceived relationship quality. Consequently, we constructed an APIMeM (Ledermann et al., 2011).

## Materials and Methods

### Participants

The sample comprised of 158 unique athletes (male *n* = 98, female *n* = 60, *M* age = 22.23, *SD* = 5.73) and their coaches (119 unique coaches; male *n* = 121, female *n* = 37, *M* age = 32.43, *SD* = 10.90) participated in this study. The sample consisted of 132 dyads who were involved in team sports and 26 dyads from individual sports. The athletes competed at amateur (*n* = 123), semi-professional (*n* = 31), or professional levels (*n* = 4), and were white (*n* = 148), black (*n* = 6), or mixed race (*n* = 4). The coaches were white (*n* = 152) or black (*n* = 6). The athletes reported a mean playing experience of 9.5 years (*SD* = 6.30), whereas the coaches reported a mean experience of 14.1 years (*SD =* 10.05). The mean relationship duration was 1.95 years (*SD* = 1.82), and the mean amount of time spent together per week was 9.5 h (*SD* = 3.10), which included time spent training, competing, and traveling.

### Measures

#### Dyadic Coping

Participants completed a coach or athlete version of the Dyadic Coping Inventory (DCI; Levesque et al., 2014) to measure dyadic coping. The DCI is a 37-item questionnaire that measures positive and negative dyadic coping. In original questionnaire, many items contained the phrase “my partner.” We replaced this term with either “my coach” or “my athlete.” Positive dyadic coping comprises of supportive (e.g., “my coach/athlete shows that he/she cares”), delegated (e.g., “When my coach/athlete feels he/she has too much to do, I help him/her out”), and common dyadic coping (e.g., “We engage in a serious discussion about the problem and think through what has to be done”). Negative dyadic coping included questions such as “my coach/athlete blames me for not coping well with stress,” “I provide support, but do so reluctantly because I think my coach/athlete should be able to cope on his/her own,” and “When I’m stressed, my coach/athlete tends to withdraw from me and does not speak to me.” All questions were answered on 5-point Likert-Type scale ranging from 1 = *never* to 5 = *very often.* Two of the original items: “We help each other relax with such things like massage, taking a bath together, or listening to music together” and “We are affectionate to each other, make love and try that way to cope with stress” were re-worded to “We help each other relax” and “We talk to show each other we care to try and cope with stress.” With a sample of 709 undergraduate students, Levesque et al. (2014) reported Cronbach alpha coefficients ranging between 0.78 and 0.85 for subscales the DCI subscales.

#### Coach–Athlete Relationship

The Coach Athlete Relationship Questionnaire (CART-Q; Jowett and Ntoumanis, 2004) assessed the athletes’ and coaches overall perceptions of relationship quality. The CART-Q is an 11-item questionnaire that measures closeness, commitment, and complementarity. All participants responded to the stem “This questionnaire aims to measure the quality and content of the coach–athlete relationship. Please read carefully the statements below and circle the answer that indicates whether you agree or disagree.” A question that assessed closeness was “I trust my coach/athlete.” A question from the commitment subscale was “I am committed to coach/athlete,” whereas “When I am coached by my coach/with my athlete, I adopt a friendly stance” was from the complementarity subscale. All questions were answered on a 7-point Likert-type scale, which ranged from 1 = *strongly disagree* to 7 = *strongly agree*. Jowett and Ntoumanis (2004) reported Cronbach alpha coefficients of 0.86 for closeness, 0.83 for commitment, and 0.78 for complementarity.

#### Primary Stress Appraisals

We used challenge and threat questions from the Stress Appraisal Measure (SAM; Peacock and Wong, 1990) to assess these primary stress appraisals. The SAM was developed outside the sport psychology literature, but is widely used among athletic populations (i.e., Gan et al., 2009; Nicholls et al., 2012, 2014). Although the SAM is usually used to measure anticipated stressors, similar to the present study, Gan et al. (2009) also employed this questionnaire to assess how athletes usually appraise stressors. Participants completed four challenge questions (e.g., “Sport has a positive impact on me,” and “I am usually excited about thinking about playing/coaching in competitions”) and four threat questions (e.g., “Competing/coaching in my sport usually makes me feel anxious” and “I usually think that the outcome of matches/competitions will be negative and that I/my athlete(s) will lose”). Questions from the SAM were answered on a 5-point Likert-type scale, which ranged from 1 = *not at all* to 5 = *extremely.* Peacock and Wong (1990) reported Cronbach alpha coefficients of 0.65, 0.73, and 0.75 for threat, along with Cronbach alpha coefficients of 0.66, 0.74, and 0.79 for challenge.

### Procedure

A university department ethics committee granted ethical approval for this study. Following ethical approval, information letters were sent to coaches and athletes. The information letter included background information on the study, requirements of the participating, and rights of all participants. If the athletes and their coaches decided to take part in the study, they signed a consent form. Coaches and athlete received an appropriately worded questionnaire pack, which contained the DCI (Levesque et al., 2014), CART-Q (Jowett and Ntoumanis, 2004), and the challenge and threat items from the SAM (Peacock and Wong, 1990). In instances where more than one athlete with same coach participated in the study, the coach was required to complete a separate questionnaire pack for each athlete he or she coached, so the information reported related to the specific coach–athlete dyad.

### Data Analysis

Preliminary data analysis comprised of screening for outliers, missing data, and univariate normality using descriptive statistics. Internal consistency was assessed using omega point estimates and bootstrapped confidence intervals, as recommended by Dunn et al. (2013). Bivariate correlations were used to explore relationships between coach variables, athlete variables, and coach to athlete variables.

**FIGURE 1:**
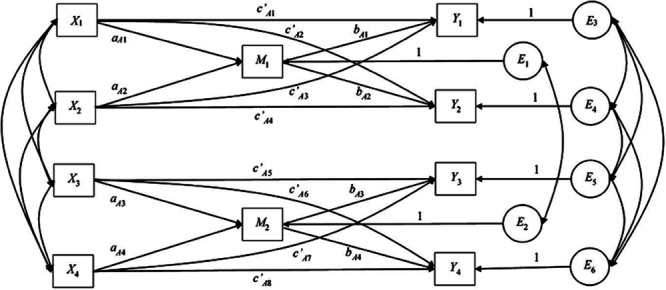
**Actor–Partner Interdependence Mediation Model showing actor effects only.** *X*_1_, coach positive dyadic coping; *X*_2_, coach negative dyadic coping; *X*_3_, athlete positive dyadic coping; *X*_4_, athlete negative dyadic coping; *M*_1_, coach relationship quality; *M*_2_, athlete relationship quality; *Y*_1_, coach challenge; *Y*_2_, coach threat; *Y*_3_, athlete challenge; *Y*_4_, athlete threat. E_1_ to E_6_, error terms.

**FIGURE 2:**
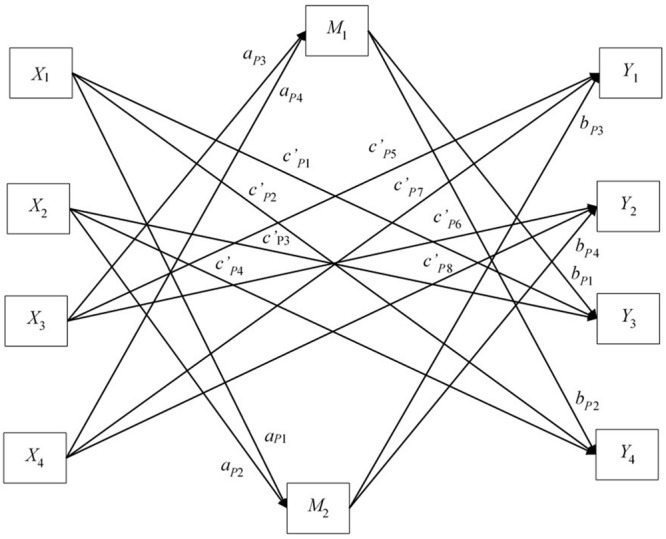
**Actor–Partner Interdependence Mediation Model showing partner effects only.** Covariances and error terms excluded to improve clarity.

Our APIMeM contained four predictor variables (*X*_1_–*X*_4_), two mediator variables (*M*_1_–*M*_2_), and four outcome variables (*Y*_1_–*Y*_4_), which were indexed as actor (*_A_*) and partner (*_P_*) depending on whether they represented an effect within a group (*_A_*) or between a group (*_P_*). There are a total of four actor *a* effects, four actor *b* effects, eight actor *c’* effects, four partner *a* effects, four partner *b* effects, and eight partner *c’* effects. For the sake of interpretation, two figures are provided to illustrate model paths; **Figure 1** shows only the actor effects and **Figure 2** shows only the partner effects. In practice, all effects are estimated within one saturated model.

## Results

Preliminary analyses revealed that less than 0.1% of data was missing and there were no outliers. Descriptive statistics, normality estimates, and omega point estimates with 95% confidence intervals for internal consistency are presented in **Table 1**. All coach and athlete subscales demonstrated acceptable univariate skewness (<2) and kurtosis (<7) with the exception of athlete negative coping, which was slightly skewed and leptokurtic. As departures from multivariate normality were to be addressed in the main, no transformation was required. Omega point estimates and confidence intervals were obtained using the MBESS package (Kelley and Lai, 2012) in R (R Development Core Team, 2012) with 1,000 bootstrap samples. All subscales comfortably exceeded the generally acceptable level of greater than 0.70.

**Table 1:** Descriptive statistics, univariate normality estimates, and omega point estimates with confidence intervals.

Variable	Mean	*SD*	Minimum	Maximum	Skew	Kurt	ω (95% CI)
**Coach**							
Positive dyadic coping	64.70	14.79	22.00	92.00	-0.26	-0.47	0.94 (0.93, 0.95)
Negative dyadic coping	15.47	5.18	8.00	39.00	1.00	1.76	0.79 (0.73, 0.86)
Relationship quality	62.56	11.68	33.00	77.00	-0.65	-0.58	0.94 (0.92, 0.95)
Challenge	17.68	2.81	5.00	20.00	-1.31	3.49	0.81 (0.74, 0.86)
Threat	8.50	3.65	4.00	20.00	0.79	0.31	0.87 (0.82, 0.92)
**Athlete**							
Positive dyadic coping	62.17	17.91	21.00	91.00	-0.53	-0.32	0.87 (0.81, 0.92)
Negative dyadic coping	16.62	5.94	8.00	53.00	2.46	10.91	0.77 (0.68, 0.83)
Relationship quality	6.17	10.36	24.00	77.00	-1.31	1.78	0.95 (0.93, 0.96)
Challenge	16.88	3.75	6.00	20.00	-1.64	2.21	0.80 (0.77, 0.83)
Threat	8.43	2.44	4.00	17.00	0.61	1.03	0.90 (0.86, 0.93)

### Correlational Analysis

Pearson bivariate correlations between variables were calculated separately for athlete and coach scores (**Table 2**) and for combined coach and athlete scores (**Table 3**). Relationship quality, indicated by the sum of closeness, commitment, and complementarity was positively associated with positive dyadic coping across each assessment, with the combined coach–athlete correlation presenting a strong relationship (*r* = 0.62, *p* < 0.01). Conversely, relationship quality was negatively associated with negative dyadic coping in all calculations, although most strongly among athletes (*r* = -0.53, *p* < 0.01). Challenge was positively associated with positive dyadic coping and relationship quality for both coaches and athletes. Threat was positively associated with negative dyadic coping but negatively associated with positive dyadic coping and relationship quality in both coaches and athletes (**Table 2**).

**Table 2:** Bivariate correlations between variables for separate coach and athlete pairings.

Variable	1	2	3	4	5
(1) Positive dyadic coping	–	0.03	0.57^∗∗^	0.10	0.09
(2) Negative dyadic coping	-0.23^∗∗^	–	-0.27^∗∗^	0.01	0.39^∗∗^
(3) Relationship quality	0.62^∗∗^	-0.53^∗∗^	–	0.27^∗∗^	-0.30^∗∗^
(4) Challenge	0.29^∗∗^	-0.24^∗∗^	0.31^∗∗^	–	-0.03
(5) Threat	-0.19^∗^	0.33^∗∗^	-0.24^∗∗^	-0.49^∗∗^	–

*Coach to coach correlations appear above the diagonal, athlete to athlete correlations appear below the diagonal.*

**Table 3:** Bivariate correlations from combined coach–athlete variables and coach to athlete pairings.

Variable	1	2	3	4	5
(1) Positive dyadic coping	0.39^∗∗^	-0.22^∗∗^	0.62^∗∗^	0.20^∗^	-0.10
(2) Negative dyadic coping	-0.01	0.11	-0.47^∗∗^	-0.13	0.35^∗∗^
(3) Relationship quality	0.19^∗^	-0.27^∗∗^	0.36^∗∗^	0.33^∗∗^	-0.30^∗∗^
(4) Challenge	0.02	-0.10	0.08	-0.01	-0.31^∗∗^
(5) Threat	0.16^∗^	0.09	-0.11	-0.13	0.23^∗∗^

*Combined coach–athlete correlations appear above the diagonal, coach to athlete correlations appear below the diagonal, Scores on the diagonal represent correlation between coaches and athletes on same construct.*

The correlations presented in **Table 3** highlight the relationship between combined coach–athlete variables, in addition to coach and athlete scores. It is noteworthy that there is a lack of significant correlations in this latter pairing. This indicates that the relationship between variables is substantively different between coaches and athletes. This point is further illustrated along the diagonal in **Table 3**. Negative dyadic coping and challenge stress appraisals from coach to athlete responses are not related.

### Actor–Partner Interdependence Mediation Modeling

We constructed an APIMeM in Mplus 7 (Muthén and Muthén, 2012) to examine the hypothesized dyadic effects of coping, relationship quality, and stress appraisal. The first step of the APIMeM was to examine the saturated distinguishable model and test all effects (Ledermann et al., 2011). Parameter estimates for this model are presented in **Table 4**. Of note, all four *a* effects, whereby dyadic coping (*X*) was posited to predict relationship quality (*M*), for coach relationship quality were significant. That is, the coach perception of relationship quality was positively predicted by both coach (*a_A_*_1_ *β* = 0.584, *p* < 0.001) and athlete (*a_P_*_3_ *β* = 0.548, *p* < 0.001) positive dyadic coping positively, but negatively predicted by coach (*a_A_*_2_ *β* = -0.303, *p* < 0.001) and athlete (*a_P_*_4_ *β* = -0.420, *p* < 0.001) negative dyadic coping. By contrast, the athlete perception of the relationship quality was not significantly predicted by coach or athlete dyadic coping. A similar pattern was observed for the *b* effects, whereby relationship quality (*M*) was posited as a predictor of stress appraisal (*Y*). Specifically, coach challenge appraisal was positively predicted by coach perception of relationship quality (*b_A_*_1_ *β* = 0.383, *p* < 0.01), and coach threat was negatively predicted by both coach (*b_A_*_2_ *β* = -0.339, *p* < 0.05) and athlete (*b_P_*_4_ *β* = -0.209, *p* < 0.05) perception of relationship quality. Athlete *b* effects were all non-significant.

**Table 4:** Unstandardized and standardized parameter estimates effects in saturated distinguishable model.

Effect	Estimate	*SE*	*p*	Standard estimate
***a* effects (*X* → *M*)**				
Coach positive coping → Coach relqual (*a_A_*_1_)	0.337	0.047	<0.001	0.584
Coach negative coping → Coach relqual (*a_A_*_2_)	-0.530	0.223	0.018	-0.303
Athlete positive coping → Athlete relqual (*a_A_*_3_)	-0.052	0.053	0.328	-0.075
Athlete negative coping → Athlete relqual (*a_A_*_4_)	-0.163	0.114	0.154	-0.081
Coach positive coping → Athlete relqual (*a_P_*_1_)	-0.023	0.038	0.551	-0.035
Coach negative coping → Athlete relqual (*a_P_*_2_)	-0.047	0.184	0.798	-0.024
Athlete positive coping → Coach relqual (*a_P_*_3_)	0.433	0.045	<0.001	0.548
Athlete negative coping → Coach relqual (*a_P_*_4_)	-0.946	0.160	<0.001	-0.420
***b* effects (*M*→ *Y*)**				
Coach relqual → Coach challenge (*b_A_*_1_)	0.138	0.042	0.001	0.383
Coach relqual → Coach threat (*b_A_*_2_)	-0.080	0.034	0.020	-0.339
Athlete relqual → Athlete challenge (*b_A_*_3_)	0.033	0.032	0.314	0.073
Athlete relqual → Athlete threat (*b_A_*_4_)	-0.005	0.036	0.895	-0.015
Coach relqual → Athlete challenge (*b_P_*_1_)	0.033	0.032	0.314	0.120
Coach relqual → Athlete threat (*b_P_*_2_)	-0.014	0.043	0.740	-0.041
Athlete relqual → Coach challenge (*b_P_*_3_)	-0.024	0.038	0.524	-0.076
Athlete relqual → Coach threat (*b_P_*_4_)	-0.044	0.022	0.046	-0.209
***c’* effects (*X* → *Y*)**				
Coach positive coping → Coach challenge (*c’_A_*_1_)	-0.030	0.020	0.126	-0.146
Coach positive coping → Coach threat (*c’_A_*_2_)	0.032	0.013	0.013	0.238
Coach negative coping → Coach challenge (*c’_A_*_3_)	0.083	0.065	0.206	0.131
Coach negative coping → Coach threat (*c’_A_*_4_)	0.115	0.055	0.037	0.280
Athlete positive coping → Athlete challenge (*c’_A_*_5_)	0.049	0.023	0.034	0.261
Athlete positive coping → Athlete threat (*c’_A_*_6_)	-0.028	0.030	0.360	-0.112
Athlete negative coping → Athlete challenge (*c’_A_*_7_)	-0.088	0.049	0.070	-0.163
Athlete negative coping → Athlete threat (*c’_A_*_8_)	0.191	0.057	0.001	0.271
Coach positive coping → Athlete challenge (*c’_P_*_1_)	-0.026	0.016	0.109	-0.167
Coach positive coping → Athlete threat (*c’_P_*_2_)	0.001	0.025	0.980	0.003
Coach negative coping → Athlete challenge (*c’_P_*_3_)	0.039	0.041	0.334	0.083
Coach negative coping → Athlete threat (*c’_P_*_4_)	0.011	0.054	0.838	0.018
Athlete positive coping → Coach challenge (*c’_P_*_5_)	0.011	0.029	0.698	0.044
Athlete positive coping → Coach threat (*c’_P_*_6_)	0.045	0.014	0.002	0.274
Athlete negative coping → Coach challenge (*c’_P_*_7_)	-0.064	0.080	0.424	-0.088
Athlete negative coping → Coach threat (*c’_P_*_8_)	-0.007	0.037	0.851	-0.015

*relqual, relationship quality.*

Direct effects (*c’*), where dyadic coping (*X*) is modeled to predict stress appraisal (*Y*) includes eight actor and eight partner effects. Four of the actor effects were statistically significant. Specifically, these were that coach threat was positively predicted by both coach positive (*c’_A_*_2_ *β* = 0.238, *p* < 0.05) and negative (*c’_A_*_4_ *β* = 0.280, *p* < 0.05) dyadic coping, athlete challenge was predicted by athlete positive dyadic coping (*c’_A_*_5_ *β* = 0.261, *p* < 0.05), and athlete threat was predicted by athlete negative dyadic coping (*c’_A_*_8_ *β* = 0.271, *p* < 0.01). Only one significant direct partner effect was observed, where athlete positive dyadic coping positively predicted coach threat appraisal (*c’_P_*_7_ *β* = 0.274, *p* < 0.01).

The total effects, total indirect effects, simple indirect effects, and direct effects are presented in **Table 5**. As expected, it is evident that the actor effects (coach = 0.454, athlete = 0.398) are substantively stronger than the partner effects (coach = 0.119, athlete = 0.268). Total direct effects accounted for 57.27% if total effect in the coach portion of the APIMeM, meaning that almost half of the effects observed were accountable for the mediation effects of relationship quality. In particular, the indirect paths from positive and negative dyadic coping to challenge appraisals accounted for a substantive proportion of this variance. Despite a similar total actor effects for the athlete, this was almost wholly (89.45%) accounted for my direct effects. Inspection of the bootstrapped confidence intervals revealed that none of the simple indirect effects were statistically significant. In summary, although the actor effects were relatively large compared to partner effects, for athletes, the relationship quality had little impact but for coaches, the mediating role of this variable was broadly as important as dyadic coping.

**Table 5:** Total effects, total indirect effects, simple indirect effects, and direct effects *c’* for distinguishable coach–athlete dyads.

Effect	Estimate	95% CI	Proportion of the total effect
**Coach actor effect**			
Total effect	0.454		
Total IE	0.194		42.73
Actor–actor IE (*a_A_*_1_*b_A_*_1_)	0.047	0.005, 0.091	10.35
Actor–actor IE (*a_A_*_1_*b_A_*_2_)	-0.027	-0.056, -0.002	5.95
Actor–actor IE (*a_A_*_2_*b_A_*_1_)	-0.073	-0.209, -0.009	16.08
Actor–actor IE (*a_A_*_2_*b_A_*_2_)	0.042	0.004, 0.139	9.25
Partner–partner IE (*a_P_*_1_*b_P_*_3_)	0.001	-0.003, 0.011	0.22
Partner–partner IE (*a_P_*_1_*b_P_*_4_)	0.001	-0.003, 0.010	0.22
Partner–partner IE (*a_P_*_2_*b_P_*_3_)	0.001	-0.016, 0.049	0.22
Partner–partner IE (*a_P_*_2_*b_P_*_4_)	0.002	-0.014, 0.034	0.44
Total direct effect	0.260		57.27
Direct effect *c’* (*c’_A_*_1_)	-0.030	-0.079, 0.023	6.61
Direct effect *c’* (*c’_A_*_2_)	0.032	-0.001, 0.065	7.05
Direct effect *c’* (*c’_A_*_3_)	0.083	-0.102, 0.276	18.28
Direct effect *c’* (*c’_A_*_4_)	0.115	0.016, 0.263	25.33
***Athlete actor effect***			
Total effect	0.398		
Total IE	0.042		10.55
Actor–actor IE (*a_A_*_3_*b_A_*_3_)	0.008	-0.029, 0.043	2.01
Actor–actor IE (*a_A_*_3_*b_A_*_4_)	-0.002	-0.043, 0.043	0.50
Actor–actor IE (*a_A_*_4_*b_A_*_3_)	-0.017	-0.089, 0.063	4.27
Actor–actor IE (*a_A_*_4_*b_A_*_4_)	0.005	-0.089, 0.098	1.26
Partner–partner IE (*a_P_*_3_*b_P_*_1_)	-0.002	-0.012, 0.003	0.50
Partner–partner IE (*a_P_*_3_*b_P_*_2_)	0.001	-0.005, 0.014	0.25
Partner–partner IE (*a_P_*_4_*b_P_*_1_)	-0.005	-0.043, 0.007	1.26
Partner–partner IE (*a_P_*_4_*b_P_*_2_)	0.002	-0.015, 0.048	0.50
Total direct effect	0.356		89.45
Direct effect *c’* (*c’_A_*_5_)	0.049	-0.009, 0.110	12.31
Direct effect *c’* (*c’_A_*_6_)	-0.028	-0.114, 0.039	7.04
Direct effect *c’* (*c’_A_*_7_)	-0.088	-0.220, 0.035	22.11
Direct effect *c’* (*c’_A_*_8_)	0.191	0.020, 0.320	47.99
**Coach partner effect**			
Total effect	0.119		
Total IE	0.042		35.29
Actor–partner IE (*a_A_*_1_*b_P_*_1_)	0.011	-0.016, 0.042	9.24
Actor–partner IE (*a_A_*_1_*b_P_*_2_)	-0.005	-0.041, 0.033	4.20
Actor–partner IE (*a_A_*_2_*b_P_*_1_)	-0.017	-0.115, 0.023	14.29
Actor–partner IE (*a_A_*_2_*b_P_*_2_)	0.008	-0.064, 0.100	6.72
Partner–actor IE (*a_P_*_1_*b_A_*_3_)	0.000	-0.010, 0.003	0.00
Partner–actor IE (*a_P_*_1_*b_A_*_4_)	0.000	-0.004, 0.007	0.00
Partner–actor IE (*a_P_*_2_*b_A_*_3_)	-0.001	-0.036, 0.014	0.84
Partner–actor IE (*a_P_*_2_*b_A_*_4_)	0.000	-0.024, 0.030	0.00
Total direct effect	0.077		64.71
Direct effect *c’* (*c’_P_*_1_)	-0.026	-0.069, 0.015	21.85
Direct effect *c’* (*c’_P_*_2_)	0.001	-0.067, 0.065	0.84
Direct effect *c’* (*c’_P_*_3_)	0.039	-0.040, 0.168	32.77
Direct effect *c’* (*c’_P_*_4_)	0.011	-0.120, 0.175	9.24
**Athlete partner effect**			
Total effect	0.268		
Total IE	0.141		52.61
Actor–partner IE (*a_A_*_3_*b_P_*_3_)	-0.011	-0.058, 0.032	4.10
Actor–partner IE (*a_A_*_3_*b_P_*_4_)	-0.019	-0.049, 0.005	7.09
Actor–partner IE (*a_A_*_4_*b_P_*_3_)	0.023	-0.067, 0.121	8.58
Actor–partner IE (*a_A_*_4_*b_P_*_4_)	0.041	-0.007, 0.124	15.30
Partner–actor IE (*a_P_*_3_*b_A_*_1_)	-0.007	-0.043, 0.007	2.61
Partner–actor IE (*a_P_*_3_*b_A_*_2_)	0.004	-0.005, 0.017	1.49
Partner–actor IE (*a_P_*_4_*b_A_*_1_)	-0.023	-0.085, 0.013	8.58
Partner–actor IE (*a_P_*_4_*b_A_*_2_)	0.013	-0.006, 0.059	4.85
Total direct effect	0.127		47.39
Direct effect *c’* (*c’_P_*_5_)	0.011	-0.060, 0.081	4.10
Direct effect *c’* (*c’_P_*_6_)	0.045	0.012, 0.085	16.79
Direct effect *c’* (*c’_P_*_7_)	-0.064	-0.289, 0.135	23.88
Direct effect *c’* (*c’_P_*_8_)	-0.007	-0.103, 0.085	2.61

*IE, indirect effect. Total effects all presented as positive for ease.*

Although partners in this model are clearly theoretically distinguishable, Ledermann et al. (2011) explain that it is necessary to test if they are empirically distinguishable. As such, paths that were theoretically justified as distinguishable (i.e., coach vs. athlete) were tested for indistinguishability to determine if the model could be simplified. To test this, we systematically imposed equal constraints on pairs of actor and partner direct effects. The results of these are presented in **Table 6**. First, we constrained comparable direct effects from dyadic coping to relationship quality. Overall model fit with each path identified in **Table 6** constrained as equal to its dyadic partner presented a model fit identifying significant misfit and therefore distinguishability (χ^2^(16) = 37.57, *p* = 0.0017, CFI = 0.939, TLI = 0.851, SRMR = 0.103, RMSEA = 0.092 [90% CI = 0.054, 0.131]). However, this was borderline. As the actor–partner effects had yielded lower chi-square values than the actor-actor effects, it was likely that these were less distinguishable. Consequently, we ran a further model constraining only these elements as equal. This resulted in excellent model fit, as one would expect: χ^2^(8) = 6.748, *p* = 0.5641, CFI = 1.000, TLI = 1.017, SRMR = 0.021, RMSEA = 0.000 [90% CI = 0.000, 0.083]. This indicates that only the actor–actor effects are empirically distinguishable, while the actor–partner effects are largely empirically indistinguishable.

**Table 6:** Tests of indistinguishability.

Constrained direct effects	χ^2^	*df*	*p*
**Actor-actor effects**			
*a* effects (*X →M*)			
*X*_1_*→M*_1_, *X*_3_*→M*_2_	2.261	1	0.1327
*X*_2_*→M*_1_, *X*_4_*→M*_2_	6.010	1	0.0142
*b* effects (*M →Y*)			
*M*_1_*→Y*_1_, *M*_2_*→Y*_3_	6.588	1	0.0103
*M*_1_*→Y*_2_, *M*_2_*→Y*_4_	2.809	1	0.0937
***c’* effects (*X → Y*)**			
*X*_1_*→Y*_1_, *X*_3_*→Y*_3_	7.328	1	0.0068
*X*_1_*→Y*_2_, *X*_3_*→Y*_4_	3.836	1	0.0502
*X*_2_*→Y*_1_, *X*_4_*→Y*_3_	5.666	1	0.0173
*X*_2_*→Y*_2_, *X*_4_*→Y*_4_	1.078	1	0.2992
**Actor-partner effects**			
*a* effects (*X →M*)			
*X*_1_*→M*_2_, *X*_3_*→M*_1_	0.213	1	0.6444
*X*_2_*→M*_2_, *X*_4_*→M*_1_	0.469	1	0.4933
*b* effects (*M →Y*)			
*M*_1_*→Y*_3_, *M*_2_*→Y*_1_	1.460	1	0.2269
*M*_1_*→Y*_4_, *M*_2_*→Y*_2_	0.438	1	0.5081
***c’* effects (*X → Y*)**			
*X*_1_*→Y*_3_, *X*_3_*→Y*_1_	1.404	1	0.2361
*X*_1_*→Y*_4_, *X*_3_*→Y*_2_	2.550	1	0.1103
*X*_2_*→Y*_3_, *X*_4_*→Y*_1_	1.772	1	0.1832
*X*_2_*→Y*_4_, *X*_4_*→Y*_2_	0.079	1	0.7793

Next, we followed the recommendations of Kenny and Ledermann (2010) by exploring the data for dyadic patterns in the APIMeM. Specifically, these could be an actor-only, couple, contrast, or partner-only pattern. A non-zero actor effect with a zero partner effect indicates an actor only pattern, whereby the partner has little influence. The couple pattern is evident when both actor and partner effects are non-zero and equal in magnitude. The contrast pattern occurs when the actor and partner effects are non-zero, equal in magnitude, but in opposing directions, and the partner-only patterns represents a non-zero partner effect with a zero actor effect. These patterns can be examined through the computation of the ratio of actor and partner effects (parameter *k*). Kenny and Ledermann (2010) recommended the computation of bootstrapped confidence intervals for *k*. The results (**Table 7**), indicate a largely actor-only affect is evident.

**Table 7:** *k* estimates with confidence intervals.

*k*	Estimate	95% CI	*P*
*ka*1 (*Pa*1 BY *M*1)	-0.155	-0.525, 0.226	0.283
*ka*2 (*Pa*2 BY *M*1)	0.307	-0.305, 2.401	0.449
*ka*3 (*Pa*3 BY *M*2)	-0.052	-0.237, 0.203	0.549
*ka*4 (*Pa*4 BY *M*2)	0.050	-0.381, 0.666	0.811
*kb*1 (*Pb*1 BY *Y*1)	-0.176	-1.179, 1.1596	0.759
*kb*2 (*Pb*2 BY *Y*2)	0.549	-0.144, 4.557	0.523
*kb3* (*Pb*3 BY *Y*3)	1.853	-1.414, 13.769	0.425
*kb4* (*Pb*4 BY *Y*4)	3.016	-8.765, 12.916	0.269

## Discussion

In this study, we assessed the relations between dyadic coping, perceived relationship quality, along with appraisals of threat and challenge among coach–athlete dyads. We also constructed an APIMeM to explore the interdependence of coach and athlete stress appraisals on perceived relationship quality and dyadic coping. The correlations revealed that on the whole, our hypotheses were supported. The actor–partner analyses indicated that relationship quality is an important mediator between dyadic coping and stress appraisal, but that the effect is much greater on coaches than athletes.

Our findings illustrate how dyadic coping relates to appraisals of threat and challenge, through the perceived relationship quality. When coach–athlete dyads report more positive dyadic coping, they experience greater satisfaction with their relationship, and consequently view stressful events as a challenge. Conversely, when coach–athlete dyads report more negative dyadic coping, they report lower relationship quality scores, and view stress as a threat. Bodenmann (2008) proposed that dyadic coping is a three-factor process, which involves interaction by both partners. One partner displays stress signals to the other partner in the form of non-verbal, verbal, or paraverbal (i.e., how we say our words spoken) communication. The other partner then attempts to understand these stress signals using passive (i.e., observing the other person), active (i.e., asking questions about the stressful situation), or interactive strategies (i.e., communicating with the other person; Berger and Bradac, 1982), and then reacts to these stress signals with dyadic coping. An athlete, for example, may display signs of stress whilst training such as being much quieter than he or she would normally be, performing at a lower level than usual, or displaying different body language, which the coach observes, before deploying dyadic coping strategies (i.e., coach helps the athlete see the stressful situation differently, by saying that it will take time for the athlete to get used to the new technique that it is normal to experience performance difficulties after making significant changes to one’s technique).

How members of the dyad respond to stress signals, using dyadic coping influences the quality of the relationship (Bodenmann, 2005). Overall, our findings provide additional support for this proposition and are an extension of previous scholarly activity (Herzberg, 2013; Rottmann et al., 2015), showing that dyadic coping is prevalent among coaches and athletes and is associated with relationship quality. Further, we extend the work of previous dyadic coping research by reporting actor–partner effects in terms of relationship quality and stress appraisals. In particular, coach perceptions of relationship quality were positively associated with their own use of positive dyadic coping and athlete’s positive dyadic coping. Conversely, coach perceptions of relationship quality were negatively predicted by their own use of negative dyadic coping and if his or her athlete engaged in this form of dyadic coping. This finding illustrates the importance of dyadic coping by the coach and athlete in how a coach may perceive his or her relationship quality. Dyadic coping by the coach or athlete appears less important in influencing relationship quality for athletes. The association between relationship quality and stress appraisals followed a similar pattern. Coach challenge appraisals were positively predicted by coach perceptions of the relationship quality, whereas coach threat appraisals were negatively associated with coach and athlete relationship quality. Athlete stress appraisals, however, were not significantly related to either the athlete’s or the coach’s perception of relationship quality. These findings infer that relationship quality is less important for athletes than coaches.

Scholars linked relationship quality with enhanced well-being (Chelladurai, 1990), performance (Jowett and Cockerill, 2003) and self-concept (Jowett and Cramer, 2010) within sport. The notion that relationship quality has a stronger relationship with appraisals among coaches than athletes is a novel finding. Lafrenière et al. (2011), for example, reported a positive association between perceptions of the coach–athlete relationship quality and happiness among athletes. Although Lafrenière et al. (2011) did not explore appraisals, emotions are generated by appraisals (Lazarus, 1999). It is therefore surprising that relationship quality and appraisals were not significant among athletes in the present study. One possible explanation is that athletes may place less importance on this relationship than coaches, and are therefore less committed to their coach. This may negate feelings of goal-directed appraisals, such as threat or challenge. Indeed, research from other domains inferred that younger people are less committed and invest less within relationships than older people (Lehmiller and Agnew, 2008), meaning they have less at stake. The mean age of the athletes in this study was 10 years younger than the mean age for coaches, which may explain our finding. Additionally, when coaches perceived their relationship was poor, positive dyadic coping was associated with higher threat and lower challenge appraisals scores. Perhaps coaches were dissatisfied with their relationship, despite investing effort into the relationship via positive dyadic coping, which resulted in the coaches viewing situations are threatening rather than challenging.

APIMeM analyses revealed that coach appraisals of threat were positively associated with coach positive dyadic coping, coach negative dyadic coping, and athlete positive dyadic coping. The relationship between negative dyadic coping and threat is in agreement with our hypotheses and previous scholarly activity (Bodenmann, 1995, 2005). The finding that coach threat appraisals was positively associated with both coach and athlete positive dyadic coping is somewhat unexpected. Existing research demonstrated that coaches experience a variety of stressors relating to poor team performance, selection, and training (Thelwell et al., 2010), so these factors may have contributed to perceived threat levels. Further, many of the coaches were involved in team sports, so although a coach may have used positive dyadic coping with an athlete in the study or vice a versa, the same coach may have engaged in negative dyadic coping with other athletes, or been on the receiving end from negative dyadic coping from many athletes. Future research could examine this finding in more detail by using a clustered approach based upon different teams and ensure that all team members participate in the research. This would provide a more accurate over view the relationship.

A limitation of this study relates to the cross-sectional nature of data collection, given that Bodenmann (2005) conceived dyadic coping as a process. It should be noted, however, that many dyadic coping studies, which were guided by the Systemic-Transactional Model and used a version of the DCI (Levesque et al., 2014) also employed cross-sectional designs (i.e., Bodenmann et al., 2011; Herzberg, 2013; Johnson et al., 2013; Landis et al., 2014). Indeed, a systematic review by Traa et al. (2015) revealed that 48% of dyadic coping studies were cross-sectional. In support of our research design, Crocker et al. (2010) argued that cross-sectional research is required when little is known about a phenomenon to guide experimental or prospective research. It would be interesting to explore dyadic coping, perceptions of relationship quality, and appraisals longitudinally in order to assess fluctuations in these constructs. An additional limitation is that the sample was dominated by male coaches and athletes. Given that scholars reported gender differences in coping with interpersonal stressors (e.g., Hoar et al., 2010) there may be gender differences in dyadic coping, which may impact perceptions of relationship quality and stress appraisals.

In order to maximize relationship satisfaction among coach–athlete dyads, coach education programs could contain training on dyadic coping, similar to Bodenmann et al.’s (2014) Couple Coping Enhancement Training (CCET). In order for athletes and coaches to benefit from this, it would be important that coaches provide training in dyadic coping to athletes, and this may have beneficial impact upon stress appraisals given the positive relationships between athlete positive dyadic coping and challenge, in addition to threat appraisals and athlete negative dyadic coping. The CCET included training on stress (i.e., understanding the causes of stress, expression of stress, impact of stress on relationships), individual coping (i.e., relaxation techniques and matching coping strategies to the stressor), dyadic coping (i.e., how to identify partner’s stress, and communicating one’s own stress, in addition to supportive, delegated, and common forms of dyadic coping), communication (i.e., identification of negative communication and how it impacts close relationships, communicating using speaker and listener rules), and conflict resolution and problem solving (i.e., six-step scheme of problem solving). In light of our findings and previous research linking appraisal training to enhanced performance (i.e., Moore et al., 2015), information on appraisal training could be included in the stress component, whereby coaches could be taught how to maximize challenge appraisals, whilst minimizing threat appraisals among their athletes. Such a framework holds promise for coaches and athletes, although this type of intervention needs testing before it is administered with athletes.

In summary, we tested an Actor–Partner Interdependence Mediation Model to account for the non-independence of dyadic data. The actor–partner analyses revealed differences between athletes and coaches. Although the actor effects were relatively large compared to partner effects, for athletes, the relationship quality had little impact but for coaches, the mediating role of this variable was broadly as important as dyadic coping. These findings provide an insight in to how coach–athlete dyads interact to manage stress and indicate that perceptions of relationship quality are of particular importance for coaches, in terms of coaches interacting with their athletes to cope with, and appraise stress. Perceptions of relationship quality appear less important for athletes. Nevertheless, there are potential benefits for both athletes and coaches by increasing positive dyadic coping, which relate to increasing the incidence of challenge appraisals, but potentially decreasing threat levels. Although increasing positive dyadic coping may enhance perceptions of relationship quality among coaches, it is likely to have less influence upon athlete perceptions of relationship quality.

## Author Contributions

AN conceptualized the study and contributed to writing the manuscript. JP performed the statistical analyses and contributed to the writing of the manuscript.

## Conflict of Interest Statement

The authors declare that the research was conducted in the absence of any commercial or financial relationships that could be construed as a potential conflict of interest.
